# Protective Role of Sphingomyelin in Eye Lens Cell Membrane Model against Oxidative Stress

**DOI:** 10.3390/biom11020276

**Published:** 2021-02-13

**Authors:** Mehdi Ravandeh, Giulia Coliva, Heike Kahlert, Amir Azinfar, Christiane A. Helm, Maria Fedorova, Kristian Wende

**Affiliations:** 1 Institute of Biochemistry, University of Greifswald, Felix-Hausdorff-Str. 4, 17489 Greifswald, Germany; hkahlert@uni-greifswald.de; 2 Leibniz-Institute for Plasma Science and Technology, ZIK Plasmatis, Felix-Hausdorff-Str. 2, 17489 Greifswald, Germany; 3 Institute of Bioanalytical Chemistry, Faculty of Chemistry and Mineralogy, Universität Leipzig, Deutscher Platz 5, 04103 Leipzig, Germany; giulia.coliva@uni-leipzig.de (G.C.); maria.fedorova@bbz.uni-leipzig.de (M.F.); 4 Center for Biotechnology and Biomedicine, University of Leipzig, Deutscher Platz 5, 04103 Leipzig, Germany; 5 Institute of Physics, University of Greifswald, Felix-Hausdorff-Str. 6, 17489 Greifswald, Germany; amir.azinfar@uni-greifswald.de (A.A.); helm@uni-greifswald.de (C.A.H.)

**Keywords:** sphingomyelin, eye lens cell membrane, oxidized lipids, aging, electrochemistry, mass spectrometry, atomic force microscopy, cold physical plasma

## Abstract

In the eye lens cell membrane, the lipid composition changes during the aging process: the proportion of sphingomyelins (SM) increases, that of phosphatidylcholines decreases. To investigate the protective role of the SMs in the lens cell membrane against oxidative damage, analytical techniques such as electrochemistry, high-resolution mass spectrometry (HR-MS), and atomic force microscopy (AFM) were applied. Supported lipid bilayers (SLB) were prepared to mimic the lens cell membrane with different fractions of PLPC/SM (PLPC: 1-palmitoyl-2-linoleoyl-sn-glycero-3-phosphocholine). The SLBs were treated with cold physical plasma. A protective effect of 30% and 44% in the presence of 25%, and 75% SM in the bilayer was observed, respectively. PLPC and SM oxidation products were determined via HR-MS for SLBs after plasma treatment. The yield of fragments gradually decreased as the SM ratio increased. Topographic images obtained by AFM of PLPC-bilayers showed SLB degradation and pore formation after plasma treatment, no degradation was observed in PLPC/SM bilayers. The results of all techniques confirm the protective role of SM in the membrane against oxidative damage and support the idea that the SM content in lens cell membrane is increased during aging in the absence of effective antioxidant systems to protect the eye from oxidative damage and to prolong lens transparency.

## 1. Introduction

The oxidation of cellular components such as lipids, DNA and proteins occurs when the level of reactive species is higher than the level of antioxidants in the cells and eventually leads to the loss of cell function [1]. Aging is a process characterized by the gradual loss of tissue and organ functions over time [2]. The age-related loss of function has been connected to the accumulation of damage induced by reactive species. In addition, oxidative stress is involved in several age-related diseases such as neurodegenerative diseases, cancer, cardiovascular diseases and cataract [3,4,5]. Sphingomyelin (SM) represents ~85% of all sphingolipids in humans and it is a structural component of cellular membranes that constitutes 10–20 mol% of total plasma membrane lipids [6,7]. However, higher concentrations of SM are found in nerve tissue, red blood cells and eye lenses [8]. The physicochemical properties of many biological membranes, including those of the human lens, change during aging [9,10], and it has been shown that the hydrocarbon chains of human lens lipids become increasingly more ordered (i.e., fewer gauche kinks and reduced lipid molecular area). The increase in membrane order correlates with an increase of the sphingomyelin fraction and a decrease in phosphatidylcholine [11,12]. There is ample evidence that the ability of the human lens to resist oxidative attack actually decreases with age because total glutathione levels drop and the important enzyme glutathione reductase becomes less stable [13,14]. The increase of oxidation-resistant SM in lens cell membranes suggest a subsequent adaptation aiming to keep the lens clear for a relatively long time and protect the eye from various oxidative stress associated diseases such as cataracts [15]. The cataract is the most common cause of blindness worldwide. It forms due to various noxae such as ultraviolet radiation, nutritional deficiencies, and aging [16]. Reactive oxygen species (ROS) including the hydroxyl radicals (^•^OH), superoxide anion (^•^O_2_^–^), and H_2_O_2_ can contribute to cataract formation. Among these, hydroxyl radicals play an important role in this cascade [17]. Biological membranes are stable but dynamic barriers that play an important role in ROS compartmentalization and related signaling events [18]. Their complexity regarding chemical composition and functionality puts a challenge on the investigation of its role.

To simplify the system, biomimetic membranes such as lipid vesicles [19,20,21,22], supported lipid bilayers (SLB) [23,24,25,26], and freestanding lipid bilayers [27] have been developed [28,29,30]. Of those, SLBs as a lipid bilayer on solid substrate are compatible to various surface-sensitive techniques such as electrochemistry, atomic force microscopy (AFM) and ellipsometry [31,32,33]. Therefore, they provide a powerful platform to study the membrane properties and interaction of reactive species with lipid bilayer [34,35,36]. Various methods have been developed for generating reactive species, such as Fenton reaction [37,38], photolysis [39,40] and cold physical plasma (CPP) [41,42]. CPP as ionized gas with temperature close to human body temperature can be produced by different sources such as plasma jets or dielectric barrier discharges [42,43], and beneficial effects have been shown in several medical applications, including wound healing [44,45], cancer treatment [46,47,48,49] and dental care [50]. In addition to its application in medicine, CPP is a promising experimental source for the production of a variety of reactive oxygen (ROS, e.g., ^•^OH, ^•^O_2_^–^, ^1^O_2,_ H_2_O_2_, ^•^O, O_3_) and nitrogen species (RNS, e.g., ^•^NO, ^•^NO_2_, ONOO^−^) [51]. The type and amount of reactive species generated by CPP is controlled by various parameters such as composition and flow rate of the feed gas, dissipated power, and electrode geometry [52,53]. Furthermore, the chemical composition of the reactive species generated by the CPP depends on the distance of the plasma source from the target and the type of target [43,54]. Various methods have been developed to study the interaction of reactive species with lipid mono- and bilayers such as infrared reflection absorption spectroscopy (IRRAS) [55], atomic force microscopy [56,57], electrochemistry [58,59,60], and mass spectrometry [61,62,63]. Scholz et al. introduced an electrochemical assay to quantify the oxidative damage of self-assembled monolayers (SAM) on gold electrodes [58]. The membrane properties can also be investigated by electrochemical techniques on a self-assembled lipid monolayer at a hanging mercury drop electrode (HMDE) [64,65]. Moreover, there are several complementary techniques to study the effect of ROS on membrane. Recently, a protocol combining electrochemistry and mass spectrometry has been proposed to monitor the interaction of reactive species with supported POPC bilayers and the protective role of lipophilic antioxidants [66]. While, electrochemical measurements provided a fast information on the residual functionality of the SLB, subsequent high-resolution mass spectroscopy (HR-MS) measurements revealed covalent changes to the molecular structure of the lipids.

In the current study, this approach was applied to shed light on the role of SM in eye lens cell membranes against oxidative damage. AFM complemented the approach, allowing the correlation of functional, chemical, and structural changes. It shows how degradation of amphiphilic molecules alters lipid self-assembly within a membrane, provides a topographic image of the supported lipid bilayer (SLB), and information on oxidative damage in PLPC/SM membranes. Several studies have reported that the amount of sphingolipids in the cell membrane of the mature mammalian eye ranges from 60% to 80% [67,68,69,70]. It was also mentioned that in cataract lens membrane, the ratio of sphingolipids is over 80% [68,71]. Therefore, in the model of the mature lens membrane, 75% sphingomyelin was chosen as the sphingolipid fraction and 25% PLPC represent phosphocholine lipids. The sphingolipid content in the eye lens membrane at birth has been reported to be approximately 30% [71]. Therefore, for the young eye lens cell membrane model 25% sphingomyelin and 75% PLPC was used. 1-palmitoyl-2-linoleoyl-sn-glycero-3-phosphocholine (PLPC (16:0/18:2)) and n-oleoyl-d-erythro sphingosylphosphorylcholine (SM (d18:1/18:1)) were selected because such phospholipids with unsaturated alkyl chains have been reported in mammalian eye lens membrane [72,73] and they are preferred targets of lipid peroxidation during oxidative stress [74]. For this purpose, three solid supported PLPC bilayers with different SM fractions were prepared to mimic the lens cell membranes of the eye during the aging process. Furthermore, a cold physical plasma jet (kINPen^®^) was used to form a mixture of biologically relevant reactive species. We could show that a higher fraction of SM resulted in a decrease in lipid oxidation products during CPP treatment; and the SLB was more stable and did not show pores. 

## 2. Materials and Methods

### 2.1. Materials

1-palmitoyl-2-linoleoyl-sn-glycero-3-phosphocholine (PLPC) and n-oleoyl-d-erythro sphingosylphosphorylcholine (SM (d18:1/18:1)) were obtained from Avanti Polar Lipids (Alabaster, AL, USA). Methyl-tert-buthylether (MTBE) and chloroform were purchased from Sigma Aldrich (Taufkirchen, Germany). Potassium ferrocyanide (K_4_[Fe(CN)_6_]), disodium hydrogen phosphate (Na_2_HPO_4_), and sodium dihydrogen phosphate (NaH_2_PO_4_) were obtained from Merck (Darmstadt, Germany). Sodium sulfate was purchased from VWR (Darmstadt, Germany). UPLC-grade methanol, acetonitrile, formic acid, and isopropanol were obtained from Biosolve BV (Valkenswaard, Netherlands). Hydrogen peroxide (30%) and 32% ammonia solutions are from Carl Roth (Karlsruhe, Germany). 50 mM buffer solution of (Na_2_HPO_4_/NaH_2_PO_4_) (pH = 7.4) was used in the experiments. All chemicals employed were used without further purification. In all solutions and experiments ultrapure water was used (resistivity: 18.2 MΩ.cm, conductivity: 0.055 μS/cm) prepared by Thermo Scientific GenPure pro water purification system (Niederelbert, Germany).

### 2.2. Plasma Source and Treatment Procedure 

A kINPen^®^ (neoplas, Greifswald, Germany) as a CPP source and argon as feed gas (3 standard litres per minute) were used for production of reactive oxygen and nitrogen species [53]. For electrochemistry and mass spectrometry, 10 mL of ultrapure water and for AFM experiments 1.5 mL of ultrapure water covering a solid supported lipid bilayer were treated with CPP. The thickness of the water layer above the supported lipid bilayer was set to 1.8 cm. The distance between plasma nozzle and water/air interface was 9 mm. 

### 2.3. Liposome Solution Preparation and Characterization

The desired amounts of PLPC, PLPC:SM (3:1 molar ratio) and PLPC:SM (1:3 molar ratio) were dissolved in chloroform. The lipid films were prepared by evaporation of solvent under constant nitrogen stream. Then the sample was placed in a desiccator for 45 min to remove the rest of solvent from the lipid film. The samples were suspended in 50 mM phosphate buffer (pH 7.4), and liposomes were prepared by sonication (tip sonicator from Hielscher Ultrasonics GmbH, Teltow, Germany) for 45 min in ice. Afterwards, the liposome solutions were centrifuged for 10 min at 14,000 rpm to remove titanium debris produced during the sonication procedure. The size distribution of the obtained SUVs was measured by dynamic light scattering (Zetasizer Nano-ZS (Malvern Instruments), Herrenberg, Germany). The final lipid concentration in the liposome solutions was 0.5 mg·mL^−1^ [75].

### 2.4. Preparation of Flat Gold Surfaces

The surface of a polycrystalline gold electrode (Ø 2 mm; Metrohm, Filderstadt, Germany) was cleaned by mechanical polishing with Al_2_O_3_ powder (Buehler, Esslingen, Germany); grain sizes 300 and 50 nm), and electrochemical cleaning by cyclic voltammetry (CV) from 0 to 1.5 V in 0.1 M H_2_SO_4_ at 0.1 V s^−1^ for about 40 cycles. Moreover, by additional 10 cycles in 0.1 M H_2_SO_4_ from 0.75 to 0.2 V at 0.1 V·s^−1^ the gold oxides were removed from the surface of the electrode [66,76].

### 2.5. Fabrication of Solid Supported Lipid Bilayers

Lipid bilayers on gold surface were formed by potential assisted vesicle fusion [75]. Briefly, the liposome solution was added to the electrochemical cell. Then, liposomes rupture was induced by cyclic voltammetry with slow potential scans (5 mV·s^−1^) between 0.4 and −0.8 V vs. Ag/AgCl for at least 4 h. After lipid bilayer formation on gold surface, the rest of liposomes was replaced by ultra-pure water for plasma treatment experiments [59,75].

### 2.6. Electrochemical Measurements

Cyclic and differential pulse voltammetric measurements were performed in 10 mM solution of K_4_[Fe(CN)_6_] in 50 mM phosphate buffer (pH 7.4). A potential range from −0.3 V to +0.7 V vs. Ag/AgCl and a scan rate of 50 mV·s^−1^ was applied (Autolab PGSTAT 20 and Eco Chemie IME 303, Metrohm AG, Filderstadt, Germany) [59]. The modified gold electrode, a platinum electrode and an Ag/AgCl (3 M KCl) electrode were used as working electrode, counter electrode, and reference, respectively. Data were recorded with NOVA 2.0 software (Metrohm AG, Filderstadt, Germany). Each lipid bilayer preparation and electrochemical experiment was repeated at least three times (*n* = 3). Data analysis was performed with OriginPro 2019 (OriginLab Cooperation, Northampton, MA, USA).

### 2.7. Lipid Bilayer Extraction

PLPC, PLPC:SM (3:1 molar ratio) and PLPC:SM (1:3 molar ratio) lipid bilayers were collected from the surface of the electrode after addition of 2 mL MTBE. After shaking the solution for 15 min, aqueous (water) and organic (MTBE) phases appeared and the MTBE phase was separated. Then, MTBE was removed under constant nitrogen stream, and the obtained lipid film was kept at −80 °C. Prior to liquid chromatography and mass spectrometry, the dried lipids were dissolved in isopropanol (final concentration was 100 nM).

### 2.8. Liquid Chromatography-Tandem Mass Spectrometry Analysis

To perform the chromatographic separation a Vanquish Focused+ UHPLC (Thermo Fisher Scientific, Bremen, Germany) system equipped with Accucore C18 column (150 mm × 2.1 mm; 2.6 µm; Thermo Fisher Scientific, Bremen, Germany) was employed. The column temperature was at 50 °C and the flow rate was 300 µL/min. Eluent A was acetonitrile:water (50:50, *v*/*v*), and eluent B was isopropanol:acetonitrile:water (85:10:5, v/v), both containing 0.1% formic acid and ammonium formate (5 mmol/L). The elution gradient was: 0–20 min ramp from 10% to 86% B, 20–22 min ramp to 86% to 95% B, 22−26 min 95% B, and the column was then re-equilibrated at 10% B for 8 min. LC was coupled on-line to Q Exactive Plus Hybrid Quadrupole-Orbitrap Mass Spectrometer (Thermo Fisher Scientific, Bremen, Germany) operated in positive ion mode. The S-lens radio frequency (RF) level was set to 35%. Capillary temperature was set to 300 °C, and the aux gas heater temperature was 370° C. Sheath, aux and sweep gas flow rates were set to 40, 10 and 1 arbitrary units, respectively. Data-dependent acquisition (DDA) settings: the full-scan mode (scan range mass to charge ratio (*m/z)* 400 to 1200) was acquired at a resolution of 140,000 (at *m/z* 200). Automatic gain control target was set to 3 E6 and maximum injection time was 100 ms. The MS/MS spectra were acquired at a resolution of 17,500 (at *m/z* 200) for top 15 most intense ions in each MS survey scan. Selected precursors were subjected higher-energy collisional dissociation (HCD) using stepped normalized collision energy (15, 20, 30), isolation width of 1.2 Da, automatic gain control target of 1 E5, maximum injection time of 60 ms, and selection intensity threshold of 3.3 E3. All spectra were acquired in profile mode. Data were analysed using Xcalibur (Thermo Fisher Scientific; version 4.0) and quantification was performed with Lipostar (Molecular Discovery; version 1.0.7). The graphs were prepared by GraphPad Prism 7 and the chemical structures of PLPC and SM were drawn by MarvinSketch© software (version 18.8.0).

### 2.9. Atomic Force Microscopy (AFM) Imaging and Analysis

Single side polished silica (100) wafers (Silicon Materials, Kaufering, Germany) were used as a support of lipid bilayer for AFM studies. The RCA-1 cleaning protocol was used for cleaning of silica substrate. Briefly, silica wafers were immersed in a mixture of 30% hydrogen peroxide, 32% ammonia solution and ultrapure water in a ratio of 1:1:5, and heated to 75 °C for 15 min. Then, the silicon wafers were washed several times with ultrapure water [77,78]. The lipid bilayer on silicon substrate was prepared by incubation of 0.5 mg/mL of liposome solution with substrate for 45 min, afterwards the remaining liposomes were removed by rinsing with buffer solution. The AFM imaging of samples in buffer solution was performed in a Bioscope Resolve by FESP-V2 cantilevers (Bruker, Raunheim, Germany) in tapping mode. Surfaces of 5 µm × 5 µm were measured from three different locations of each lipid bilayer. The Nanoscope analysis software version 1.9 was used to process the images.

## 3. Results and Discussion

### 3.1. Role of SM in Size and Stability of Liposomes 

Liposomes were prepared by sonication and their sizes were determined by dynamic light scattering (DLS) confirming the formation of small unilamellar vesicles (SUV) with an average radius of 76.3 ± 1.2 nm, 75.8 ± 0.7 nm and 96.1 ± 1.2 nm for PLPC, PLPC:SM (3:1) and PLPC:SM (1:3), respectively (Table 1). The results indicated that at a lower fraction of SM, the size of liposomes is almost similar to PLPC but there is an increase in radius of liposomes with 75% SM. Since similar sonication energy (60-Hz repetition frequency and a duty cycle of ~50%) was used for all liposomes, it seems this energy is not enough to make smaller size of SM-rich liposomes (PLPC:SM (1:3)) due to increase in number of hydrogen bonds between the head groups within the lipid bilayer [79]. A higher sonication energy was avoided to minimize the oxidation of lipids during liposome preparation.

The stability of liposomes was studied by potential assisted vesicle fusion [80]. The formation of a lipid bilayer on the gold surface was monitored by cyclic voltammetry (CV) based on oxidation and reduction peaks of ferrocyanide/ferricyanide as a redox probe. In this one-electron redox reaction, the ferrocyanide ion [Fe(CN)_6_]^4−^ is a reductant and the ferricyanide ion [Fe(CN)_6_]^3−^ is an oxidant [81]. The chemical equation for this reaction is as follows:

(1) $${\left[\mathrm{Fe}{\left(\mathrm{CN}\right)}_{6}\right]}^{4-}⇆\;{\left[\mathrm{Fe}{\left(\mathrm{CN}\right)}_{6}\right]}^{3-}\;+\;e^{-}.$$

**Figure 1:**
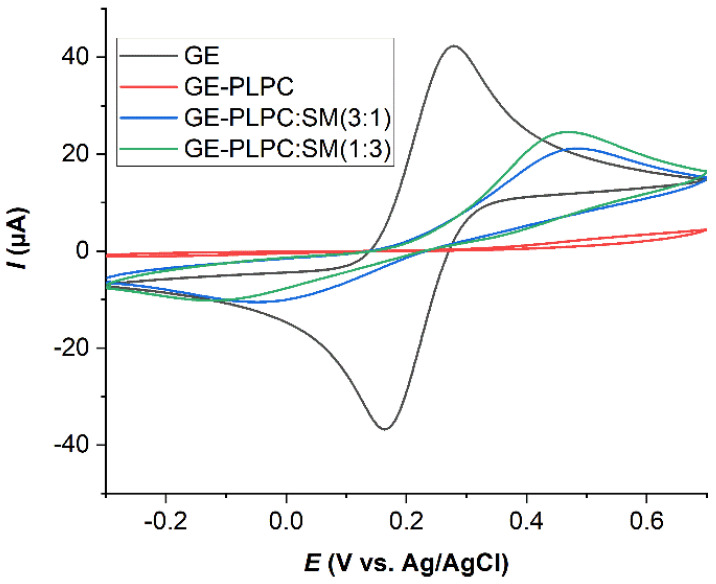
Cyclic voltammograms (CVs) of 10 mM K_4_[Fe(CN)_6_] in 50 mM phosphate buffer solution (scan rate 50 mV·s^−1^) for bare gold electrode (GE, black curve) and after formation of PLPC (PLPC, red curve), PLPC:SM (3:1, blue curve), and PLPC:SM (1:3, green curve) lipid bilayers, respectively.

The oxidation/reduction current of the redox probe reflects non-covered areas of the electrode and the resulting electron transfer between this area and the redox probe. When the electrode surface is completely covered with lipid bilayer, no electrons can be exchanged between the gold electrode and the redox probe, i.e., the oxidation/reduction of the redox probe is blocked. The results (Figure 1) indicate a complete coverage of the gold electrode with PLPC bilayer. The rupture of PLPC liposomes is consistent with the almost complete disappearance of the oxidation and reduction peaks of the redox probe. However, for PLPC/SM lipid bilayers the peak current was increased compared to PLPC lipid bilayers confirming the higher stability of PLPC/SM liposomes, which led to a lower coverage of the electrode surface. The role of SM lipids in increasing the mechanical stability of liposomes [79] is also confirmed by the electrochemistry results.

### 3.2. Effect of ROS on Model Eye Lens Cell Membrane in Presence and Absence of SM: Electrochemistry

Differential pulse voltammetry (DPV) was used to monitor the protective role of SM against oxidative stress in SLBs inspired by the eye lens cell membrane (Figure S1). Supported PLPC lipid bilayers with different fraction of SM were treated for 30 min by reactive species generated by CPP. The normalized recovery of oxidation peak current of redox system after treatment of PLPC-lipid bilayers with different fraction of SM was calculated with the following equation:

*A* = (*I*_t_ − *I*_cov_)/*I*_g._ (2)

where *I*_t_ (µA) and *I*_cov_ (µA) are the peak currents of redox probe after and before plasma treatment of lipid bilayers, and *I*_g_ (µA) is the peak current of redox probe at the bare gold electrode.

**Figure 2:**
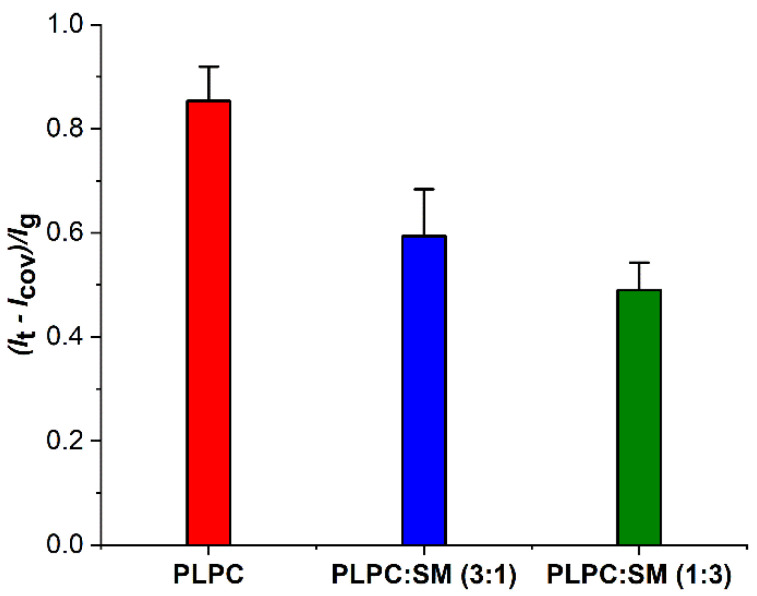
Normalized recovery peak currents of ferrocyanide/ferricyanide redox system determined by differential pulse voltammetry of lipid bilayers on gold electrodes before (*I*_cov_) and after 30 min plasma treatment (*I*_t_) (*n* = 3). Varied is the SM fraction in PLPC bilayers. Each system is normalized with respect to the peak current of the redox system for the bare gold electrode *I*_g_.

The highest normalized recovery of peak current (Figure 2) of redox system (*A*_PLPC_) was recorded for plasma treated PLPC lipid bilayer. The high peak current indicated that the molecules of the redox probe are capable of migrating and diffusing through the lipid bilayer and reaching the gold electrode. This indicates substantial damage of the lipid bilayer and possible pore formation. In contrast, the PLPC/SM-lipid bilayers show a decrease in normalized recovery peak current of redox system (*A*_PLPC:SM_), indicating that SM molecules protect lipid bilayers during oxidative stress. The protection in presence of SM could be due to structural interaction of SM and PLPC molecules. Since there is an amide and hydroxyl group in the head groups of SM for accepting and donating hydrogen, hydrogen bonding between PLPC and SM lipids in membrane occurs. Such attractive force reduces the penetration of reactive species into the membrane and decreases the oxidation damage in the alkyl chains [71,82].

The protective effect of SM in model membranes was calculated by using the following (Equation (3)):

% Protective effect = [(*A*_PLPC_ − *A*_PLPC:SM_)/*A*_PLPC_] × 100. (3)

where *A*_PLPC_ and *A*_PLPC:SM_ are the normalized recovery peak currents of redox system obtained after plasma treatment of lipid bilayer in absence and presence of SM, respectively. The electrochemical results indicated a protective effect of 30% and 42% in presence of 25% and 75% SM in lipid bilayer, respectively. These observations indicate the ability of the SM to protect lipid bilayer during oxidative stress. 

### 3.3. Effect of ROS on Model Eye Lens Cell Membrane in Presence and Absence of SM: HR-Mass Spectrometry

**Figure 3:**
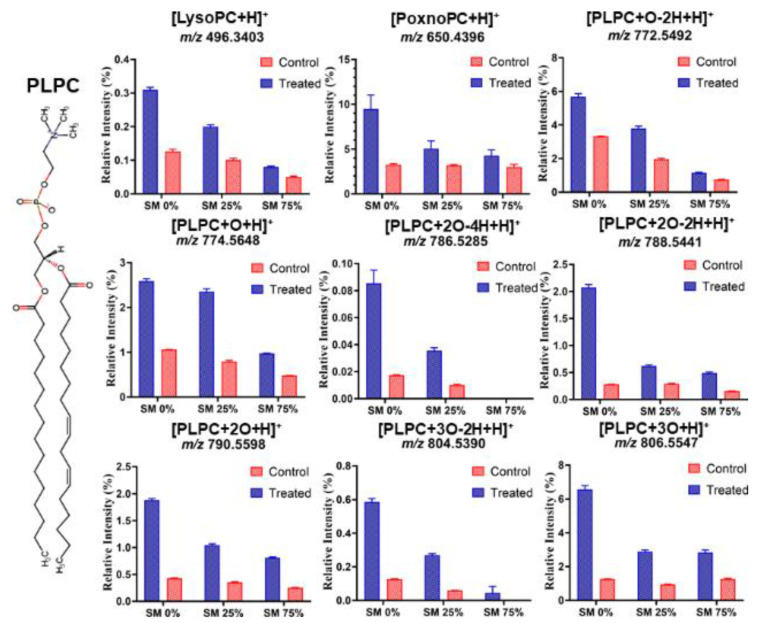
The relative intensity for different PLPC oxidation products obtained from lipid bilayers with different SM fraction after 30 min plasma treatment (Treated) and after auto-oxidation (Control) (*n* = 3). Data were obtained by HR-mass spectroscopy. The relative intensity of each lipid peroxidation product (LPP) is calculated as the peak area of LPP divided by the peak area of unmodified PLPC in the same sample. Note that the different graphs have different scaling of the *y*-axis. 1-Palmitoyl-2-hydroxy-sn-glycero-3-phosphocholine and 1-palmitoyl-2-(9′-oxo-nonanoyl)-sn-glycero-3-phosphocholine were presented as LysoPC and PoxnoPC, respectively.

While electrochemistry was used to measure the oxidative damage over the entire lipid membrane, subsequent high-resolution mass spectroscopy experiments revealed changes at the molecular level due to the impact of ROS. A significant increase in the occurrence of oxidation products was observed for PLPC and SM as identified by tandem mass spectrometry. Predominantly, various PLPC peroxidation products were detected (Table 2). Among those, the signals at *m/z*, 496.3403, and 650.4396 in positive mode were attributed to formation of 1-palmitoyl-2-hydroxy-sn-glycero-3-phosphocholine (LysoPC 16:0, “LysoPC”) and 1-palmitoyl-2-(9′-oxo-nonanoyl)-sn-glycero-3-phosphocholine (PoxnoPC 16:0_9:0 <oxo>, “PoxnoPC”), respectively. PoxnoPC as a PLPC derived aldehyde was the most abundant peroxidation product after CPP treatment. The number of lipid fragments observed (Figure 3) illustrate that the formation of PLPC oxidation products correlates inversely with the fraction of SM in the lipid bilayer and the relative abundance of PLPC oxidation products decreased with increasing fraction of SM in lipid bilayer. The protective effect of SM in PLPC bilayers also reduced the auto-oxidation of the control samples. However, the oxidation products differed from the plasma-treated ones due to the different oxidation process by ambient oxygen.

**Figure 4:**
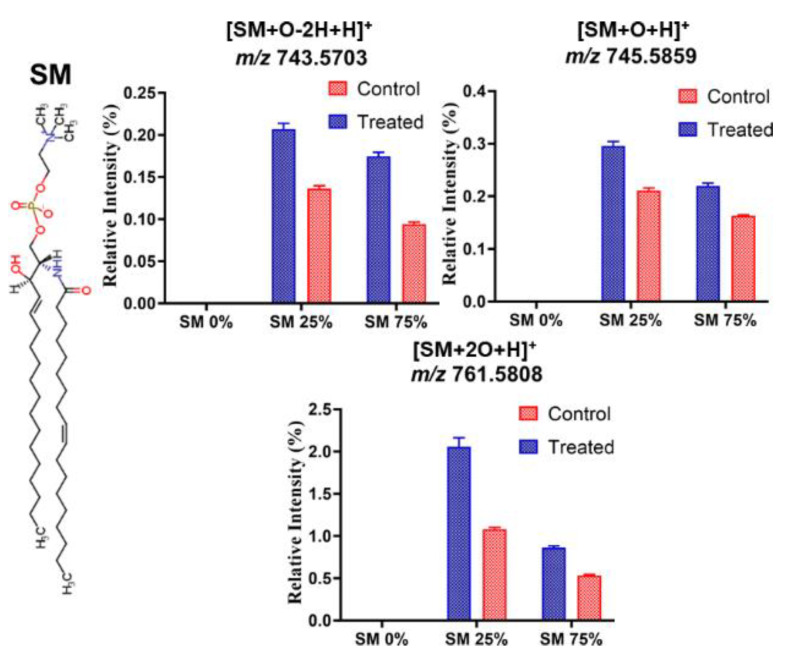
The relative intensity for different SM oxidation products obtained from lipid bilayers with different SM fraction after 30 min plasma treatments (Treated) and after auto oxidation (Control) (*n* = 3). Relative intensity of each lipid peroxidation product (LPP) is calculated as the peak area of LPP divided by the peak area of unmodified SM in the same sample. Note that the different graphs have different scaling of the *y*-axis.

In addition, the effect of ROS on SM molecules in PLPC/SM lipid bilayers was studied (Figure 4). The peaks at *m/z*, 743.5703, 745.5859, and 761.5808 in positive mode were attributed to addition of keto (SM + O − 2H), hydroxyl (SM + O), and hydroperoxyl (SM + 2O) groups to SM molecules after plasma treatments of PLPC/SM SLBs. No significant difference in lipid aldehyde formation from SM lipids was observed before and after plasma treatments of PLPC/SM lipid bilayers. The relative abundance of SM-derived oxidation products is much lower than found for PLPC. This shows the protective role of SM not only for PLPC but also for SM molecules in lipid bilayers. The suggested mechanism is the formation of a hydrogen bonding network in the head group region of the membrane in presence of SM molecules, thus limiting the access of reactive species to the alkyl chains within the lipid bilayer. In addition, the double bond in SM is in *trans* configuration, and with an α-positioned electron-drawing hydroxyl group less reactive as the aliphatic double bond(s) in fatty acid chains like in the PLPC [74]. 

**Figure 5:**
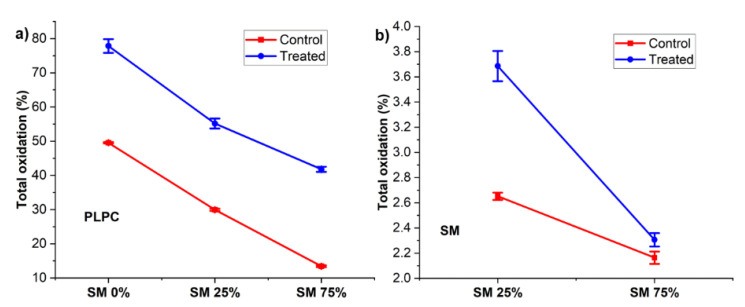
Total oxidation of PLPC (**a**) and SM (**b**) after 30 min plasma treatment of lipid bilayers with different SM fraction (indicated “Treated”) and without treatment (indicated “Control”) (*n* = 3). Total oxidation is defined as the sum of lipid peroxidation product (LPP) peak areas quantified in each sample relative to the peak area of non-oxidized parent lipid. Note that the different graphs have different scaling of the *y*-axis.

To quantify the damage of the lipids on a molecular level, we defined total oxidation as the sum of lipid peroxidation product (LPP) peak areas relative to the respective peak area of non-oxidized parent lipid of the same sample. Total oxidation versus bilayer composition shows the relationship between lipid peroxidation products (LPP) formation rates and SM ratio in supported lipid bilayers (Figure 5). The highest total oxidation was observed in SLB formed from PLPC only. The total oxidation of PLPC (Figure 5a) decreased monotonously with increasing SM content of the bilayer (78%, 55%, and 42%, respectively). The protective effect of SM for PLPC was calculated with Equation (4).

% Protective effect = [(*T*_PLPC_ − *T*_PLPC:SM_)/*T*_PLPC_] × 100 (4)

where *T*_PLPC_ and *T*_PLPC:SM_ are the total oxidation of PLPC, and PLPC with different SM fraction, respectively.

The protective effect increased with SM fraction (29% and 46% for lipid bilayers with 25% and 75% SM, respectively). The total oxidation of SM was lower than that of PLPC. This was expected from the low relative intensity of the LPPs derived from SM molecules (Figure 4). The dependence of the protective effect on SM fraction in PLPC bilayers is in agreement with electrochemical results (30% and 42% for lipid bilayers with 25 and 75% SM, respectively). Electron paramagnetic resonance (EPR) studies on similar set up in our previous work indicated that the hydroxyl radical, superoxide anion and hydrogen peroxide are the main reactive species produced in aqueous phase during plasma treatment [66]. Interestingly, these reactive species play a significant role in cataract formation in eye [17]. Several reports indicated that hydrogen peroxide does not generate a direct oxidative damage in lipid bilayer [66,83]. Therefore, short-lived reactive species play the dominant role in PLPC oxidation. Furthermore, Coliva et al. showed a similar protection effect of SM, especially concerning the dependence of LPP formation rates on molar fraction of SM in liposomes [59]. Borchman and Yappert reported a linear relationship between sphingolipids content and hydrocarbon chain conformational order. When the trans-rotamers in the alkyl chains of the lipids increase, the lipid bilayers may be more closely packed leading to an increased van der Waals attraction. In contrast, in a disordered lipid bilayer the percentage of gauche-rotamers increases [71]. 

### 3.4. Effect of ROS on Model Eye Lens Cell Membrane in Presence and Absence of SM: Atomic Force Microscopy

**Figure 6:**
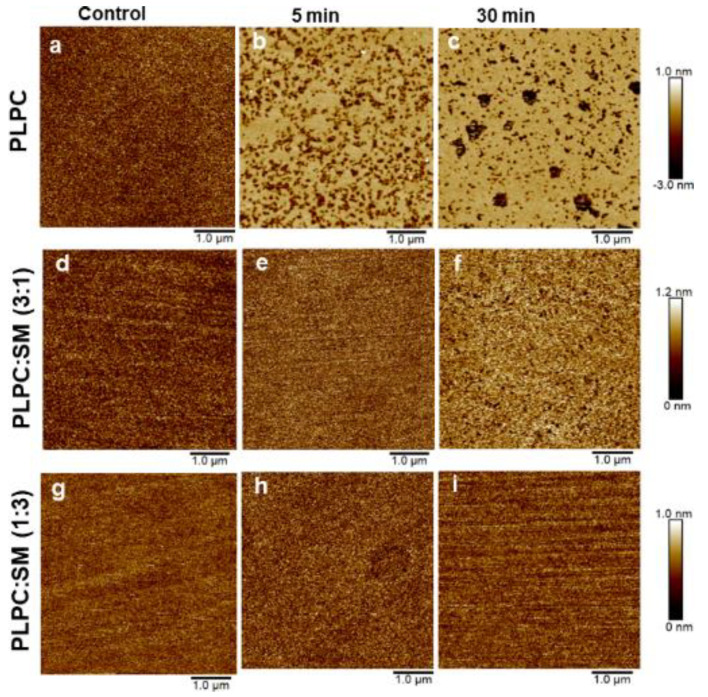
AFM images (5.0 × 5.0 µm^2^) of supported PLPC bilayers with different SM fractions before (left) and after plasma treatment for different duration (centre 5 min; right 30 min). Measurements were performed in 50 mM phosphate buffer solution at room temperature. Note the different height scale; the assignment of the height scales is (**a**, **d**, **e**, **g**, **h**, **i**: 0 to 1 nm), (**b**, **c**: −3 to 1 nm) and (**f**: 0 to 1.2 nm).

Mass spectrometry results showed that PLPC was substantially more oxidized by plasma-derived ROS than SM. By imaging the supported lipid membrane with AFM, corresponding changes in the membranes physical structure were searched. The topographic images of substrate (Figure S2) and supported lipid bilayers with different fraction of SM before and after plasma treatments were recorded (Figure 6a–i) in 50 mM phosphate buffer solution. Here, a silicon substrate was used because the dimensions of the gold electrode were not suitable for the AFM. Topographic images of lipid bilayers before plasma treatment (control) confirmed the homogeneous coverage of silicon substrate by the model lipid bilayers (Figure 6a,d,g). The change of substrate can affect the morphology of the inner leaflet of the lipid bilayer and the amount of water between the substrate and the lipid bilayer. However, since both the silicon and polycrystalline gold are hydrophilic surfaces, and in addition, the plasma treatments were performed from above and the first interaction site of the reactive species is the outer leaflet of the lipid bilayer, it is assumed that the change of substrates has no significant effect on the protective role of SM in this study. For PLPC bilayers pore formation and bilayer degradation were observed after 5 min, which got more pronounced after 30 min (Figure 6b,c). However, if the bilayer consisted of 25% SM and 75% PLPC in 25% of investigated area of SLB, partial bilayer degradation was observed after 30 min of plasma treatment of PLPC/SM bilayers (Figure 6f). Importantly, no change was observed in topographic images of SLBs with 75% SM even after 30 min (Figure 6g–i).

**Figure 7:**
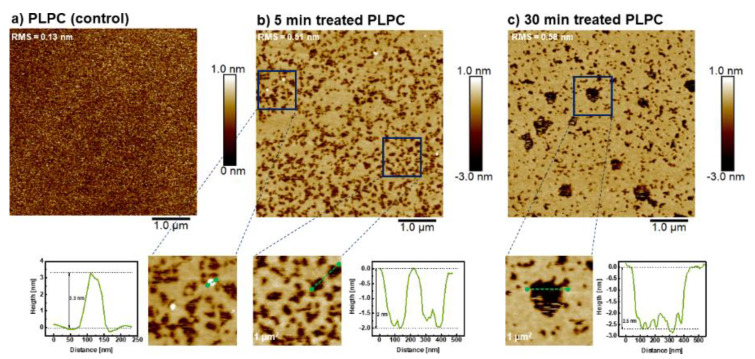
AFM images of supported PLPC bilayers before (**a**), after 5 min; and (**b**) after 30 min; (**c**) plasma treatment. Bottom row: The images are magnifications of the indicated areas. Histograms along the green lines show the height of the protrusions (left), and the increasing depth of the pores with increasing treatment time. Experimental conditions as in Figure 6.

**Figure 8:**
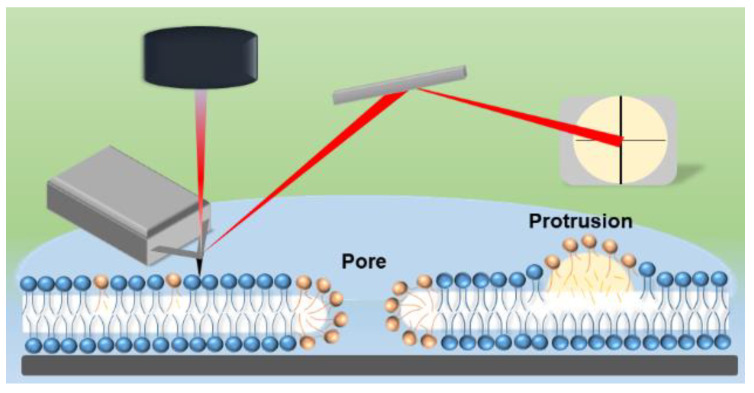
Schematic of the experimental AFM setup and supported PLPC bilayers after plasma treatment. Shown are both a pore and a protrusion. Brown color indicates fragmented or oxidized PLPC molecules, blue color the native state.

The roughness (rms) of PLPC lipid bilayer was 0.13, 0.51 and 0.58 nm before and after 5 and 30 min plasma treatments, respectively. CPP derived reactive species yielded to the formation of an inhomogeneous surface (Figure 7b,c). Interestingly, pores were observed. The pores grew (Figure 7b,c) with longer treatment time, and both diameter and depth increased. In addition, several white patches were observed at the surface of lipid bilayer, indicating protrusion formation with the height of 3.3 nm in PLPC lipid bilayer. Qualitatively, protrusions were more often observed after short-time treatments, similar to experiments reported by Tero et al. [56]. The mechanism of formation and positive curvature of protrusion can be explained by accumulation of fatty aldehydes released from lipids in hydrophobic core of membrane during attack of reactive species, such as ^•^OH and ^•^O_2_^–^ to lipid bilayer and formation of truncated-chain lipid (Figure 8). The formed protrusion can be considered as a precursor for pore formation during long-term treatments [56,57]. Notably, no significant pore formation and lipid degradation was observed in PLPC/SM (3:1) bilayers after 5 min treatment. Only slight lipid degradation with pore diameter of less than 100 nm was observed in 25% of investigated area of lipid bilayer (Figure S3). After 30 min treatment, the surface roughness was somewhat increased to 0.17 nm. PLPC/SM (1:3) supported lipid bilayers remained unchanged even after 30 min plasma treatment. To confirm the presence of a bilayer at the surface of the silicon and to exclude confusion due to similar roughness of lipid bilayer and substrate, rupture and pore formation in the SLB were induced by exposure to air and subsequently monitored (Figure S4). The AFM images complement and confirm the protective effect of SM in PLPC bilayers observed with electrochemistry and mass spectroscopy. Pore formation occurs when a high amount of lipid aldehydes such as PoxnoPC is produced during the oxidation of the lipid bilayer, which was only achieved during treatment of the PLPC lipid bilayer without SM as demonstrated by mass spectrometry (cf. Figure 3 and Figure 5) [84]. The absence of membrane degradation and pore formation in the presence of SM within the PLPC bilayer during the AFM studies is attributed to the fact that less oxidation occurred. The effects of CPP treatment of PLPC bilayers observed with AFM (pores and increased roughness) explain the increased peak current observed with electrochemistry. 

## 4. Conclusions

In the present study, the protective role of a higher fraction of SM in a model eye lens cell membrane against oxidative stress was studied on a macroscopic (by electrochemistry), molecular (by HR-mass spectrometry), and on nm (by AFM) level. PLPC bilayers with three different fractions of SM were investigated. CPP-derived reactive species were used to mimic the main reactive species in the eye such as ^•^OH, ^•^O_2_^–^_,_ and H_2_O_2_, especially during cataract formation. The results provide some insights into the mechanism of oxidative damage in human eye lens cell membrane and show the protective role of SM. The suggested molecular mechanism is consistent with literature (increasing the membrane cohesion due to hydrogen bonding of SM head groups with other lipid head groups) [71]. The dependence of membrane protection on SM fraction is consistent with the idea about the molecular mechanisms which the aging eye uses to keep lens transparency and to protect the eye against penetration of reactive species and oxidative stress. Note that the aging eye lacks a strong antioxidative system. Furthermore, the results indicate that a high amount of lipid aldehydes in a model membrane is necessary for pore formation. However, it should be considered that increasing the conformational order of hydrocarbon chains may have negative effects on membrane ion channels and the function of membrane proteins. The role of SM in the function of transmembrane proteins and ion channels in model human eye lens will be the subject of our future investigations.

## Figures and Tables

**Table 1:** Radius of PLPC liposomes with different SM molar fraction determined by DLS.

Liposomes	Mean Radius (nm)	Standard Deviation (nm)
100 mol% PLPC	76.3	1.2
75% mol% PLPC + 25% mol% SM	75.8	0.7
25% mol% PLPC + 75% mol% SM	96.1	1.2

**Table 2:** PLPC peroxidation products after 30 min plasma treatments of lipid bilayers, observed by HR-MS.

PLPC Peroxidation Product	*m/z* (Positive Mode)
[LysoPC + H]^+^	496.3403
[PoxnoPC + H]^+^	650.4396
[PLPC + O − 2H + H]^+^	772.5492
[PLPC + O + H]^+^	774.5648
[PLPC + 2O − 4H + H]^+^	786.5285
[PLPC + 2O − 2H + H]^+^	788.5441
[PLPC + 2O − 2H + H]^+^	790.5598
[PLPC + 2O − 2H + H]^+^	804.5390
[PLPC + 3O + H]^+^	806.5547

## Data Availability

Data will be available on request.

## Supplementary Materials

The following are available online at https://www.mdpi.com/2218-273X/11/2/276/s1, Figure S1: Differential pulse voltammograms of 10 mM K_4_[Fe(CN)_6_] in 50 mM phosphate buffer at bare gold electrode (GE) and before and after 30 min plasma treatments of PLPC lipid bilayers with different fractions of SM; Figure S2: AFM image (5.0 × 5.0 μm^2^) of silicon substrate after RCA cleaning; Figure S3: AFM images of supported PLPC:SM (3:1) bilayers after 30 min plasma treatment. Bottom row: The images are magnifications of the indicated areas. Histograms along the black lines demonstrate the depth of the pores. Experimental conditions as in Figure 6; Figure S4: Fig. S4. AFM images of supported PLPC:SM (1:3) bilayers after exposure to air. Bottom row: The images are magnifications of the indicated areas. Histograms along the green line demonstrate the depth of the pores. Experi-mental conditions as in Figure 6.
