# Supplementary material for: Protective Role of Sphingomyelin in Eye Lens Cell Membrane Model against Oxidative Stress
Source: Biomolecules. 2021 Feb 13;11(2):276. doi: 10.3390/biom11020276 (PMC7918908; doi:10.3390/biom11020276)
Supplement: Supplementary file 1 [file biomolecules-11-00276-s001.pdf]

Protective role of sphingomyelin in eye lens membrane model against oxidative stress

Mehdi Ravandeh, Giulia Coliva, Heike Kahlert, Amir Azinfar, Christiane A. Helm, Maria Fedorova, Kristian Wende

Supplementary information

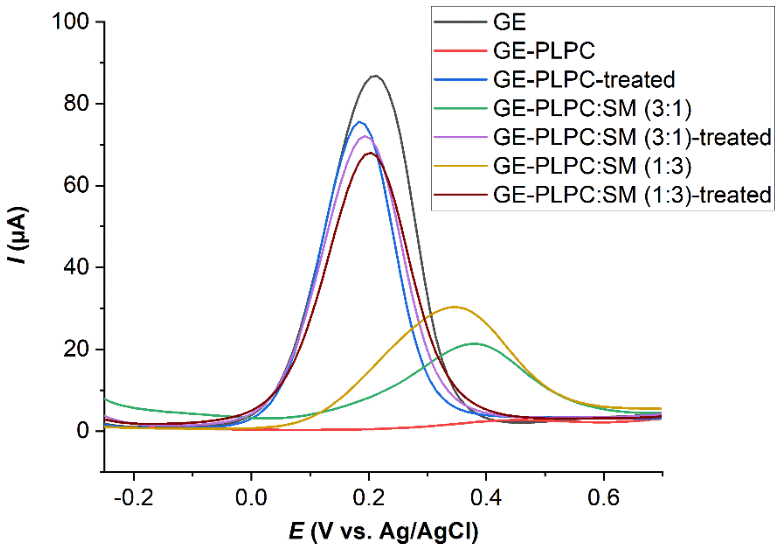

Fig. S1. Differential pulse voltammograms of 10 mM $K_4[Fe(CN)_6]$ in 50 mM phosphate buffer at bare gold electrode (GE) and before and after 30 min plasma treatments of PLPC lipid bilayers with different fractions of SM

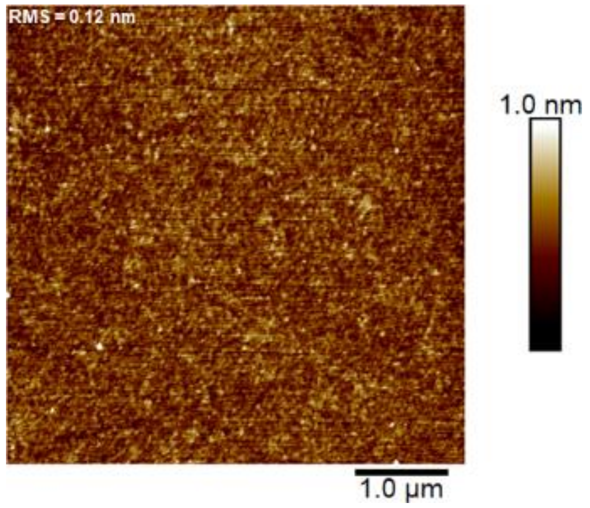

Fig. S2. AFM image (5.0 x 5.0 µm²) of silicon substrate after RCA cleaning

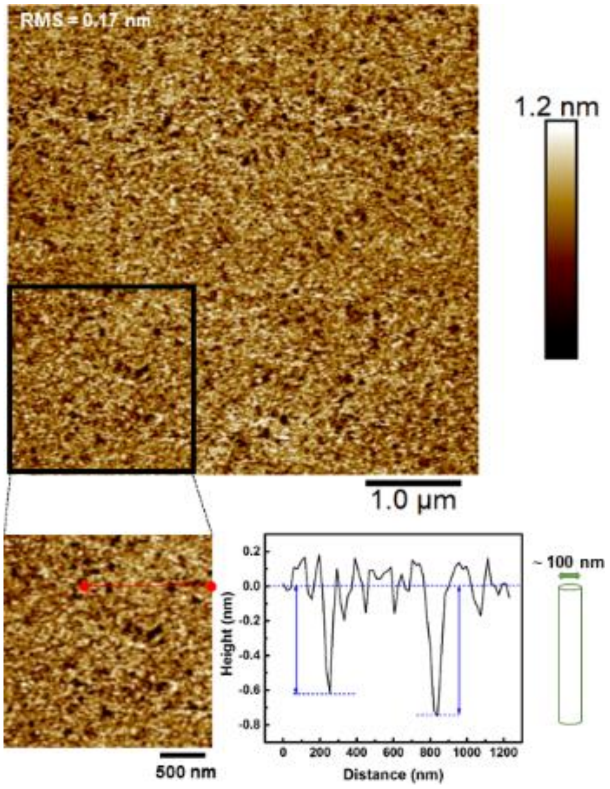

Fig. S3. AFM images of supported PLPC:SM (3:1) bilayers after 30 min plasma treatment. Bottom row: The images are magnifications of the indicated areas. Histograms along the black lines demonstrate the depth of the pores. Experimental conditions as in Figure 6.

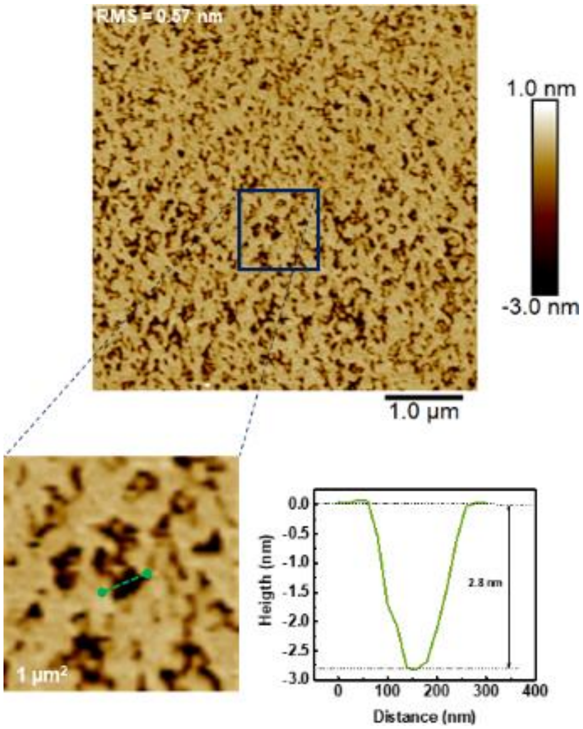

Fig. S4. AFM images of supported PLPC:SM (1:3) bilayers after exposure to air. Bottom row: The images are magnifications of the indicated areas. Histograms along the green line demonstrate the depth of the pores. Experimental conditions as in Figure 6.
